# Global and local priming in a multi-modal context

**DOI:** 10.3389/fnhum.2022.1043475

**Published:** 2023-02-28

**Authors:** Alexandra List

**Affiliations:** Department of Psychology and Neuroscience Program, Hamilton College, Clinton, NY, United States

**Keywords:** global, local, vision, audition, cross-modal processing, priming

## Abstract

Perceptual information can be processed at many different scales, from featural details to entire scenes. Attentional selection of different scales has been studied using hierarchical stimuli, with research elucidating a variety of biases in local and global attentional selection (due to, e.g., stimulus properties, brain injury, and experience). In this study, the emphasis is on biases produced through recent experience, or level-specific priming effects, which have been demonstrated within both the visual and auditory modalities. Namely, when individuals attend to local information, they are subsequently biased to attend locally (and similarly so with global attention). Here, these level-specific priming effects are investigated in a multi-modal context to determine whether cross-modal interactions occur between visual and auditory modalities during hierarchical processing. Specifically, the study addresses if attentional selection of local or global information in the visual modality subsequently biases auditory attentional selection to that level, and vice versa (i.e., level-priming). Though expected identity priming effects emerged in the study, no cross-modal *level-priming* effects manifested. Furthermore, the multi-modal context eliminated the well-established within-modality level-specific priming effects. Thus, though the study does reveal a multi-modal effect, it was not a level-based effect. Instead, paradoxically, the multi-modal context eliminated attentional scope biases (i.e., level-priming) within uni-modal transitions. In other words, when visual and auditory information are equally likely require attention, no persistence emerges for processing local or global information over time, even within a single modality.

## 1. Introduction

Our perceptual environment can be appreciated at many different scales. Visually, individuals can attend to an entire scene, objects within a scene, parts of objects and even object surface and textural qualities. The ability to adjust attentional scope has been studied using hierarchical figures in which local elements and global configurations can be independently manipulated (e.g., Navon, 1977; Kinchla and Wolfe, 1979). For example, local Es can be arranged to create a global H, and a person can flexibly identify the information at either level (local or global). In his influential report, Navon (1977) argued that participants show global precedence, wherein global information processing is prioritized over local. However, various later studies have shown that attentional scope biases are more flexible, and shift depending on stimulus parameters. For example, attentional biases to local or global information can vary depending on the absolute size of the hierarchical stimuli (local biases are more likely with larger stimuli; Kinchla and Wolfe, 1979; Lamb and Robertson, 1990), stimulus eccentricity in the visual field (global biases are more likely with more peripheral stimuli; Lamb and Robertson, 1988), and the density (global biases are more likely with denser local elements; Martin, 1979) or number (local biases are more likely with fewer local elements; Kimchi and Palmer, 1982) of local elements.

Attentional biases to local and global information has also been shown to depend on interhemispheric processing balance (see Ivry and Robertson, 1998 for a broad survey), perhaps most convincingly from studies of brain injured individuals. Specifically, right-hemisphere injuries produce a local bias and left-hemisphere injuries produce a global bias (e.g., Delis et al., 1986; Lamb et al., 1988, 1989; Robertson et al., 1988). Rafal and Robertson (1995) even argued that right-hemisphere local biases are likely contributors to hemi-spatial neglect, further exacerbating a rightward spatial bias by limiting patients’ abilities to expand their attentional window. Indeed, Bultitude et al. (2009) showed that prism adaptation, a method more commonly used to alleviate lateralized spatial biases in hemi-spatial neglect (e.g., Rossetti et al., 1998; Bultitude and Rafal, 2010), increased global processing in individuals with right temporal-parietal brain injuries. These studies of brain injured individuals provide support for the notion that the two hemispheres contribute complementarily in controlling attentional scope.

Not only do stimulus attributes and functional inter-hemispheric balance contribute to attentional scope, but so does recent experience. In healthy individuals, how someone has deployed their attention in one moment will impact their ensuing attentional scope (e.g., Ward, 1982; Robertson, 1996; Filoteo et al., 2001; List et al., 2013). Without an incentive otherwise, when individuals attend to global information, they are subsequently biased to (again) attend to global information. Similarly, attending to local information will subsequently bias attention to local information. These effects are described as level-priming, which Robertson (1996) attributed to an *attentional persistence.* Critically, level-priming is independent of identity or response priming, because it occurs whether or not a repetition of target shape or response also occurs (Robertson, 1996; also see Filoteo et al., 2001). Furthermore, level-priming is also robust to changes of stimulus location or surface attributes (Lamb and Robertson, 1988; Robertson, 1996) or to absolute stimulus size (e.g., Kim et al., 1999). Thus, level-priming has been well-isolated from other priming effects, suggesting that the scope of attentional selection is indeed what is being primed. Robertson (1996) argued that attentional persistence only arises when hierarchical parsing is necessary, in which case the attentional selection process leaves a trace, which then biases subsequent selection.

**FIGURE 1:**
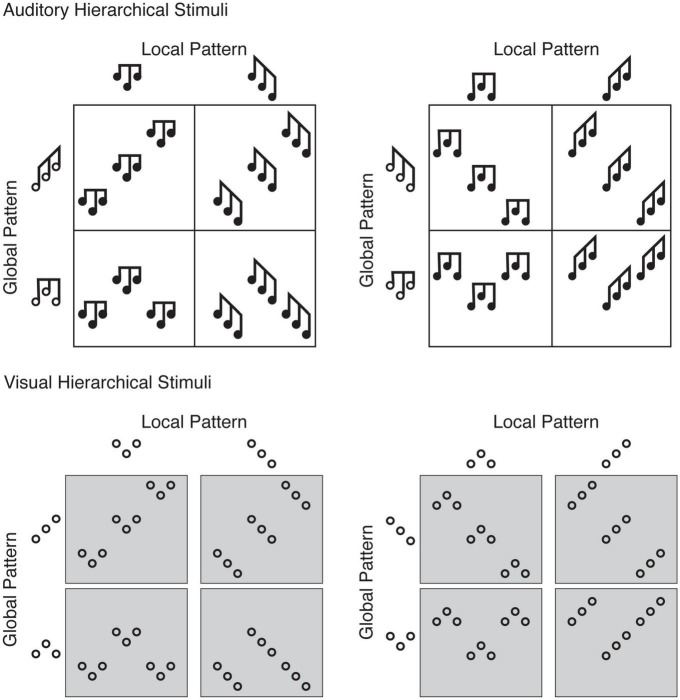
The 16 hierarchical stimuli, created by the factorial combination of rising-rising and rising-falling patterns, with falling-rising and falling-falling patterns, and hierarchical level (global and local). For each participant, because their response mapping was counter-balanced, one participant’s target pattern (e.g., a rising-rising global pattern is a target for those with rising-rising and rising-falling assignments) was a distracter pattern for another participant (the same rising-rising global pattern is a distracter for those with falling-rising and falling-falling assignments, which would be presented locally). **(Top)** For the auditory hierarchical stimuli, the black notes depict individual tones, where the horizontal axis shows time (the leftmost occurs first and proceeds left to right) and the vertical axis shows frequency (the lowest corresponds to F#_3_ and highest to A#_4_). Each local pattern is a three-tone sequence repeated three times to produce a nine-tone global pattern. If a participant were assigned a response mapping of falling-rising and falling-falling patterns, they should respond “falling-rising” when the two leftmost stimuli are presented (falling-rising pattern presented at the local level) as well as when the two bottom right stimuli are presented (falling-rising pattern presented at the global level). **(Bottom)** For the visual hierarchical stimuli, “rising” refers to a southwest-to-northeast left-to-right relationship between neighboring circles, and “falling” refers to a northwest-to-southeast left-to-right relationship between neighboring circles. As with the auditory stimuli, if a participant were assigned a response mapping of falling-rising and falling-falling patterns, they should respond “falling-rising” when the two leftmost stimuli are presented (falling-rising pattern presented at the local level) as well as when the two bottom right stimuli are presented (falling-rising pattern presented at the global level). For those more familiar with visual hierarchical letter stimuli, the analogy is presenting the letters A and E at one level, and S and H at another level. Participants respond to, for example, A and S as target patterns, whether presented at the local or global level.

Though most research on attention to hierarchical information has been conducted in the visual modality, various studies have shown that attention to different stimulus scopes also occurs in audition (e.g., Justus and List, 2005; Sanders and Poeppel, 2007; List and Justus, 2010; Ouimet et al., 2012). In auditory studies, as in visual ones, local stimulus patterns are repeated to create an overall global pattern. For example, in Figure 1, the top left hierarchical pattern represents a three-element “falling-rising” pattern repeated three times to create a global “rising-rising” pattern (imagine time elapsing on the *x*-axis and frequency on the *y*-axis, as in musical notation). Using such auditory hierarchical stimuli, attentional persistence occurs independently of target pattern, response and absolute scope repetition (Justus and List, 2005; List and Justus, 2010). Because attentional persistence to scope manifests for both vision and audition, one question is whether attentional persistence to a hierarchical level can occur across modalities. In other words, might attending to global auditory information bias an individual toward global visual information, and vice versa? Similarly, might attending to local information in one modality prime subsequent local processing in the other? In one study, Bouvet et al. (2011) showed that *unimodal* auditory and visual biases in attentional scope were correlated in individuals. Nevertheless, no study has directly assessed trial-by-trial cross-modal priming, which more directly addresses a potentially shared (or interactive) scope selection mechanism across vision and audition.

There is already evidence that attention operates across the auditory and visual modalities for spatial attention to locations (but not scope; Driver and Spence, 1998a,b). For example, Spence and Driver (1997) showed that exogenous spatial auditory cues influenced visual discrimination (but not vice versa). Their later work extended these findings to also reveal bi-directional auditory-visual inhibition of return effects (Spence and Driver, 1998; Spence et al., 2000). Other research has further delineated certain limits on cross-modal attention effects, especially for endogenous attention (e.g., Ward et al., 2000; Soto-Faraco et al., 2005; Prime et al., 2008; Ahveninen et al., 2019; though see Spence and McDonald, 2004). Studies on brain-injured individuals have demonstrated attentional independence in the auditory and visual systems by, for instance, dissociating auditory and visual spatial attention deficits (extinction and hemi-spatial neglect; e.g., Sinnett et al., 2007; Barrett et al., 2010; cf., Rapp and Hendel, 2003; Brozzoli et al., 2006; Jacobs et al., 2012). Despite demonstrations that auditory-visual spatial attention *can* be dissociated, it yet remains unknown whether the process of attending to local or global information interacts across modalities, as it can, especially, in certain exogenous situations (Spence and Driver, 1998; Spence et al., 2000; Spence and McDonald, 2004). Therefore, although research has not yet determined whether a cross-modal shared or interactive mechanism might exist for attentional scope, it is at least plausible that attentional selection of scope could operate across the visual and auditory modalities in a multi-modal context.

Therefore, in the current experiment, visual and auditory hierarchical stimuli were intermixed to test the hypothesis that the adopted attentional scope (local or global) in one modality (visual or auditory) would prime individuals to persist at that scope (local or global) in the other modality (auditory or visual). Critically, target level (local or global) and modality (auditory or visual) were unpredictable from one trial to the next. Additionally, by using analogous visual and auditory hierarchical stimuli, participants were tasked with identifying a pattern regardless of its level or modality (Figure 1). Each hierarchical stimulus was either a nine circle (visual) or tone (auditory) stimulus arranged such that each local pattern (composed of three circles or tones, respectively) was repeated three times and organized to form a global pattern. One additional important benefit of using these stimulus sets is that, unlike commonly-used alphanumeric stimuli (e.g., Navon, 1977), both the local and global patterns require grouping (List et al., 2013). Whether auditory or visual, local and global stimuli were three-element patterns (Figure 1). Participants could therefore respond to their two assigned target patterns independent of modality and hierarchical level. Should participants show a level-specific priming effect from vision to audition, or vice versa, independent of any response or target priming, this would support a shared (or at least interactive) attentional mechanism for selecting auditory and visual scope.

## 2. Materials and methods

### 2.1. Participants

As in previous studies using these auditory hierarchical stimuli (e.g., Justus and List, 2005; List and Justus, 2010), right-handed participants reporting 5 or more years of musical experience were recruited. Of the 48 who volunteered, 24 reached the practice criteria described below (13 women; 11 men; *M* = 19.96 years, *SD* = 1.40). All participants were undergraduate students who were compensated financially or with course extra credit. All participants provided written informed consent before participating (Hamilton College IRB# SP14-112).

### 2.2. Stimuli

#### 2.2.1. Auditory stimuli

Auditory hierarchical stimuli were as in Justus and List (2005), Experiment 2. Each 100-ms tone had 10-ms on and off ramps, comprised five 1/n amplitude harmonics, with fundamental frequencies in nine whole-steps ranging from F#_3_-A#_4_. Stimuli were presented at ∼72 dB SPL through Sennheiser HD280 headphones during the practice and experiment.

Hierarchical stimuli were created by sequencing nine tones without inter-stimulus intervals (Figure 1, top). Each local pattern comprised three tones presented in a falling-rising, falling-falling, rising-falling, or rising-rising sequence. Each global pattern comprised three local patterns presented in a falling-rising, falling-falling, rising-falling, or rising-rising sequence. As is shown in the top of Figure 1, a factorial combination of (falling-rising, falling-falling) by (rising-falling or rising-rising) by level (global, local) resulted in eight auditory hierarchical stimuli. In this way, participants are always only able to accurately identify one of their two assigned target patterns (either falling-rising and falling-falling, or rising-falling and rising-rising) at the local or global level. The distractor pattern (rising-falling or rising-rising, or falling-rising or falling-falling, respectively) occurs necessarily at the other level. By counter-balancing target patterns across the final group of 24 participants, the same stimulus serves as a local target trial for one group of participants and a global target trial for another group of participants.

#### 2.2.2. Visual stimuli

Visual hierarchical stimuli were as in List et al. (2013); unfilled stimulus set; Figure 1, bottom. Black visual hierarchical stimuli were centered on a white background, and comprised nine circle outlines (0.6^°^-diameter; 0.1^°^ linewidth) spanning a maximum of 7.2^°^ × 7.2^°^ for a whole nine-circle hierarchical figure, with local patterns spanning 1.9^°^ × 1.9^°^ maximum. A black filled circle (0.2^°^-diameter) served as fixation.

As in the auditory hierarchical stimuli, nine elements were arranged to create the visual hierarchical stimuli (Figure 1, bottom). Each local pattern comprised three circles presented in a falling-rising [∨], falling-falling [\], rising-falling [∧], or rising-rising [/] sequence (where the spatial relation between two circles is described as rising, a southwest to northeast direction, and falling, a northwest to southeast direction). Each global pattern comprised three local patterns presented in a falling-rising, falling-falling, rising-falling, or rising-rising sequence. As is shown in Figure 1 (bottom), a factorial combination of (falling-rising, falling-falling) by (rising-falling or rising-rising) by level (local, global) resulted in eight visual hierarchical stimuli.

### 2.3. Procedure

Participants were seated ∼57 cm from a 1,920 × 1,080 resolution monitor, running at 60 Hz. Each participant was assigned two target patterns, either rising-rising and rising-falling, or falling-falling and falling-rising (Figure 1). Each pattern was depicted as a triplet on response box keys, and participants were instructed to respond as quickly and accurately with their right and left index fingers (pattern-side mappings were counter-balanced across the final group of 24 participants). Participants reported which of their two target patterns was presented, *regardless of the level at which it occurred (global or local), or in which modality it occurred.* This is referred to as a divided-attention task in the literature (e.g., Lamb and Robertson, 1989; Hübner et al., 2007), because participants must identify one of their two target patterns without knowing prior to stimulus presentation whether it will be presented globally or locally, or auditorily or visually.

Because the auditory task is more challenging for participants to learn than the visual task, participants were familiarized with the auditory task first. They were presented with auditory examples of each target triplet alone (e.g., falling-falling and falling-rising) at a fast and a slow rate, and were given unlimited time to respond by pressing the buttons. They were then presented with eight randomly interleaved trials to further practice the task and response mapping.

Participants were then shown a visual depiction of the full array of hierarchical stimuli (akin to Figure 1) and were explicitly shown their target pattern in each of the stimuli. In order to continue to the experiment, participants were required to reach a minimum of 14/16 trials correct within six practice blocks. If participants reached criterion performance in the auditory practice, practice with the visual stimuli alone followed, and if they reached the same criterion responding to visual hierarchical stimuli, then the multi-modal auditory-visual practice followed. The 24 participants who reached the criteria to participate in the experiment completed a mean of 4.1 (*SD* = 1.5) auditory, 1.8 (*SD* = 1.3) visual and 2.3 (*SD* = 1.1) multi-modal practice blocks. Due to experimenter error, four participants’ unimodal auditory and visual practice data are missing. Participants were encouraged to ask questions between practice blocks, and to focus on responding both as accurately and as quickly as possible.

In the experiment, participants completed six blocks of 65 trials apiece, with self-paced breaks between blocks. In each block, 64 trials were sequenced so that each trial (according to its target pattern, target level and target modality) followed each other trial type once to balance priming repetition and changes. Because there were two target patterns, two levels and two modalities, eight trial types resulted which followed each of the eight trial types once (8^2^ = 64 trials). However, because the first trial is not subject to priming itself, it was repeated at the end to include it in the priming analyses (hence, 65 trials per block). Each of the 24 final participants completed six distinct fixed trial orders, and block order was varied using a partial Latin-squares design.

**FIGURE 2:**
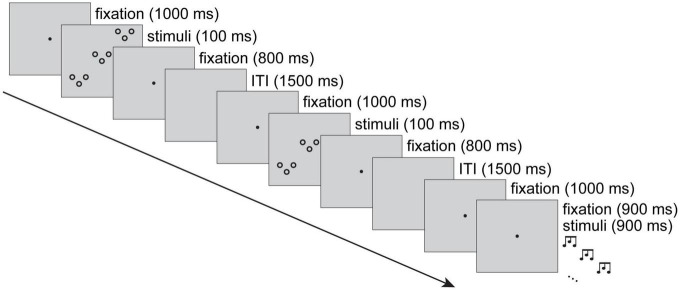
The experimental procedure displayed with a sequence of three trials. Visual and auditory trials were intermixed, with 1.5-s inter-trial intervals (ITIs). In the figure, the first trial shows a visual rising-rising global pattern and falling-rising local pattern. The second trial shows a visual rising-falling global pattern and a falling-rising local pattern. The third trial shows an auditory falling-falling global pattern and a rising-falling local pattern (while a simultaneous visual fixation is on the screen). Trials were coded according to modality, modality priming, target priming, level, and level priming. Thus, for a participant assigned falling-rising and falling-falling target patterns, in the examples depicted, accurate responses would be falling-rising, falling-rising and falling-falling. The second trial (subject to priming from the first) would contribute to the visual, within-modality, same-target, local, same-level condition, and the third trial (subject to priming from the second) would contribute to the auditory, across-modality, different-target, global, different-level condition.

Figure 2 shows an example sequence of three trials. Each auditory trial began with a central fixation dot that was presented for 1.9 s. After 1 s of fixation, a 900-ms hierarchical auditory stimulus was presented. Visual trials also began with 1 s of fixation, followed by a visual hierarchical stimulus for 100 ms, and fixation for 800 ms. Blank and silent 1.5-s inter-trial intervals (ITIs) separated all trials. Responses could occur any time from stimulus presentation until the next trial began (i.e., within 2,400 ms of stimulus onset).

Trials were coded for modality and target level, and to enable analysis of the priming effects, trials were coded according to transitions between N and N-1 target patterns, target levels, distractor pattern and modality. In Figure 2, assuming a participant is assigned falling-rising and falling-falling target patterns, they would respond falling-rising on the first trial, falling-rising on the second trial and falling-falling on the third trial. In terms of priming, the second trial is an example of within-modality (visual → *visual*), same-target pattern (i.e., same-response), same-level (local → *local*) and different-distractor pattern (rising-rising → rising-falling). In terms of priming, the third trial is an example of across-modality (visual → *auditory*), different-target pattern (i.e., different-response), different-level (local → *global*) and same-distractor pattern (rising-falling → rising-falling) trial. The design specifies the current trial’s modality (visual, auditory), target level (global, local), as well as its relation to the previous trial: modality priming (same, different), target priming (same, different) and level priming (same, different).

### 2.4. Data analysis

**FIGURE 3:**
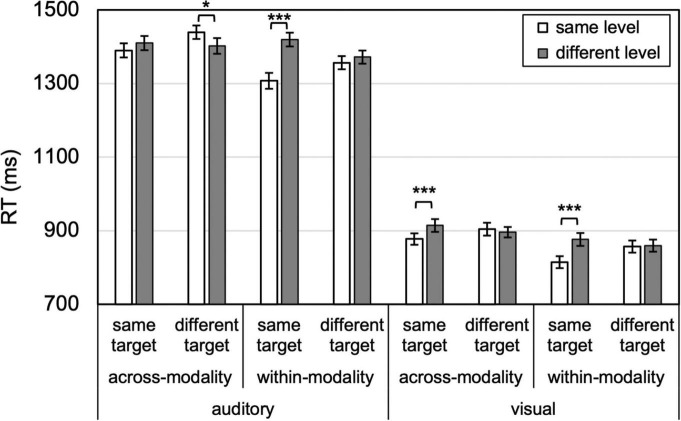
Attentional persistence to level is absent (for different targets, no benefit for same-level compared to different-level), whereas identity priming is more prevalent (for same targets, compare same- to different-level). Error bars reflect *SE*s adjusted for within-subjects comparisons, *p < 0.05, ***p ≤ 0.001.

**FIGURE 4:**
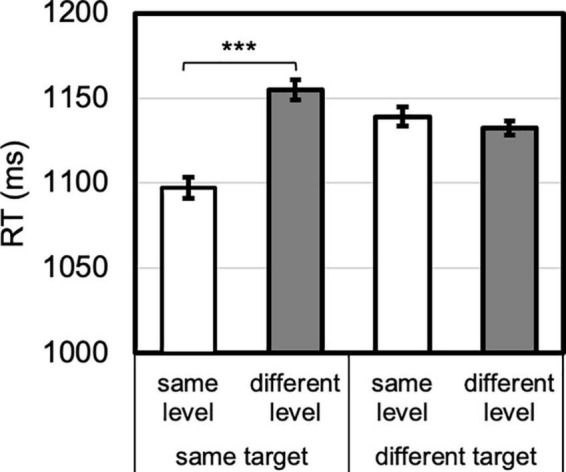
Level priming by target priming interaction. Identity priming was present (same vs. different level for same target), whereas level-priming was not (same vs. different level for different target). Error bars reflect *SE*s adjusted for within-subjects comparisons, ***p < 0.001.

**FIGURE 5:**
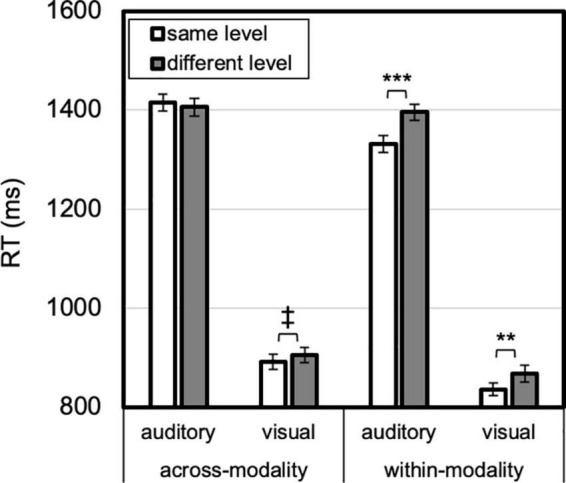
Modality by modality priming by level priming. A within-modality same-level benefit (vs. different-level) was greater for auditory than visual targets. A trend for a visual across-modality same-level benefit was present. Error bars reflect *SE*s adjusted for within-subjects comparisons, ^‡^*p* < 0.10, **p ≤ 0.01, ***p ≤ 0.001.

**FIGURE 6:**
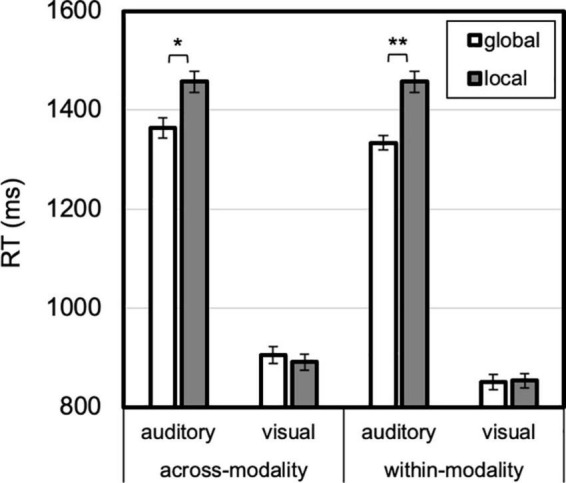
Level by modality priming by modality. An auditory global (vs. local) advantage was larger after modality repeated than when modality changed. Error bars reflect *SE*s adjusted for within-subjects comparisons, *p < 0.05, **p ≤ 0.001.

**FIGURE 7:**
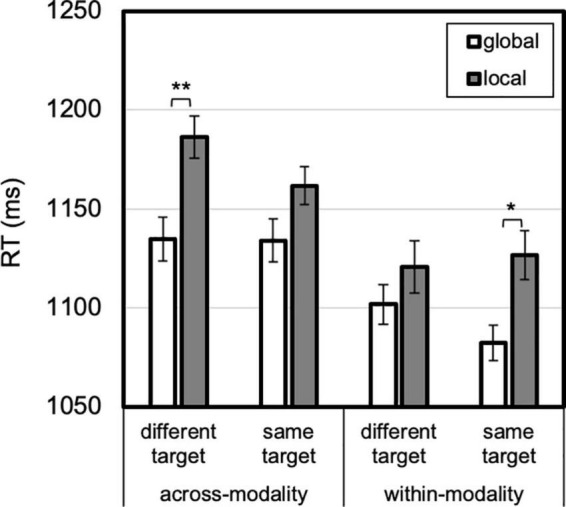
Level by modality priming by target priming. A global advantage was present for different targets after modality changed and for repeated targets after modality repeated. Error bars reflect *SE*s adjusted for within-subjects comparisons, *p < 0.05, **p ≤ 0.001.

To demonstrate level-specific attentional persistence independent of target and response priming, it is critical to compare certain conditions *a priori* (as in, e.g., List and Justus, 2010; List et al., 2013). Namely, level-specific priming is demonstrated by showing that same-level responses are facilitated relative to different-level responses, *when the target and response change*. Otherwise, the priming effect would be conflated with target (and/or response) priming. For example, to claim true auditory level-specific priming of vision, a reliable difference would need to manifest between the same- and different-level responses in the across-modality, visual, and different-target condition. Identity priming, on the other hand, is measured by comparing performance on same- and different-level trials for the *same* target pattern. For identity priming, the target pattern (and response) are held constant, and the comparison is between repeated and changed hierarchical level. Therefore, eight planned paired-samples *t*-tests were conducted for same vs. different level, for auditory trials, within or across modality, and for visual trials, within or across modality (Figure 3). Effect sizes (as Cohen’s *d*) and Bayes factors (*K*) are also reported for these analyses. The omnibus analysis and follow up analyses are depicted in Figures 4–7, and the ANOVA table is provided in the Supplementary material.

## 3. Results

Accurate trials’ (*M* = 88.4%, *SD* = 4.4; excludes both misses and errors, as well as trials following misses or errors for priming analyses) response times (RTs) were trimmed, removing outliers ±3 *SD*s, and submitted to planned paired comparisons (see section “2.4 Data analysis” above). For completeness, an omnibus repeated-measures ANOVA was also conducted with modality (auditory, visual) × modality priming (same, different) × level (local, global) × level priming (same, different) × target pattern priming (same, different) as factors. The priming factors reflect the coding of trial N, relative to trial N-1 (Figure 2).

### 3.1. Cross-modal level-priming

The primary findings are illustrated in Figure 3: no level-specific priming occurred, within or across modalities. In different visual target trials, no same- vs. different-level benefit was found within-modality or across modalities, *t*s < 1, *K*s > 3 (moderate evidence for null), nor was it present for auditory within-modality trials, *t* < 1, *K*s > 3 (moderate evidence for null), and an opposite (same > different-level) effect emerged for auditory across-modality, 37 ms, *t*(23) = 2.10, *p* = 0.047, Cohen’s *d* = 0.43, *K* = 0.09 (anecdotal evidence for difference). However, this latter result did not survive Bonferroni (*p* < 0.00625) or Bonferroni-Holm correction for multiple comparisons, whereas the following three effects did. Despite a lack of level-specific priming, identity priming emerged in three cases: Visual within-modality, 62 ms, *t*(23) = 3.907, *p* = 0.001, Cohen’s *d* = 0.81, *K* = 0.02 (very strong evidence for difference); visual across-modality, 37 ms, *t*(23) = 4.107, *p* < 0.001, Cohen’s *d* = 0.84, *K* = 0.01 (very strong evidence for difference); and auditory within-modality, *t*(23) = 4.365, *p* < 0.001, Cohen’s *d* = 0.89, *K* = 0.008 (extreme evidence for difference). These robust identity priming effects provide confidence that participants were engaged in the task, show that the experimental design was rigorous enough to detect priming effects, and rule out potential RT ceiling and floor limits in detecting level-priming effects. For the auditory across-modality trials, no reliable identity priming emerged, *t*(23) = 1.344, *p* = 0.192, Cohen’s *d* = 0.28, *K* = 2.74 (anecdotal evidence for null).

### 3.2. Omnibus ANOVA

In the omnibus ANOVA (see Supplementary Table 1 for full results), four main effects emerged. Participants responded 512 ms slower to auditory than visual targets, *F*(1, 23) = 50.372, *p* < 0.001, η*_*p*_*^2^ = 0.94. Participants were also 46 ms slower to respond when modality switched rather than repeated, *F*(1, 23) = 39.709, *p* < 0.001, η*_*p*_*^2^ = 0.63. Overall, RTs were 36 ms faster to global than local targets, *F*(1, 23) = 4.329, *p* = 0.049, η*_*p*_*^2^ = 0.16. Lastly, participants’ RTs were 25 ms faster for same-level compared to changed-level, *F*(1, 23) = 17.391, *p* < 0.001, η*_*p*_*^2^ = 0.43. No main effect was found for target priming, *F*(1, 23) = 2.408, *p* = 0.13, η*_*p*_*^2^ = 0.10. All the reliable main effects, barring Level, remained even when adopting a strict Bonferroni or Bonferroni-Holm correction for multiple comparisons.

The omnibus ANOVA also revealed six higher-order interactions that are described below, as well as follow-up paired *t*-tests. In support of the reported planned comparisons above, an overall target priming by level priming interaction emerged, *F*(1, 23) = 23.324, *p* < 0.001, η*_*p*_*^2^ = 0.50 (Figure 4). By comparing same- vs. different-level RTs, no level-priming occurred when the target changed, 7 ms (different faster than same), *t* < 1, Cohen’s *d* = 0.19, whereas identity priming did occur when the target repeated, 58 ms, *t*(23) = 5.593, *p* < 0.001, Cohen’s *d* = 1.14 (Figure 4).

A two-way interaction between modality priming and level priming, *F*(1, 23) = 24.485, *p* < 0.001, η*_*p*_*^2^ = 0.52, was qualified by three-way interaction between modality, modality priming and level priming, *F*(1, 23) = 4.496, *p* = 0.045, η*_*p*_*^2^ = 0.16 (Figure 5). When modality repeated, both auditory and visual targets were faster for same- than different-level, auditory: 64 ms, *t*(23) = 4.107, *p* < 0.001, Cohen’s *d* = 0.84; visual: 32 ms, *t*(23) = 2.807, *p* = 0.01, Cohen’s *d* = 0.57. When modality changed, however, visual responses showed a trend for benefit for same vs. different-level, 14 ms, *t*(23) = 1.991, *p* = 0.06:, Cohen’s *d* = 0.41, whereas auditory did not, 9 ms (different faster than same), *t* < 1, Cohen’s *d* = 0.17. Thus, a benefit for level-repetition (with identity and level-priming conflated) was only evident for within-modality transitions.

A two-way interaction between modality and level, *F*(1, 23) = 17.249, *p* < 0.001, η*_*p*_*^2^ = 0.43, was qualified by three-way interaction between modality, modality priming and level, *F*(1, 23) = 6.156, *p* = 0.021, η*_*p*_*^2^ = 0.21 (Figure 6). A 76-ms global advantage was present in auditory trials, *t*(23) = 3.121, *p* = 0.005, Cohen’s *d* = 0.64, but not visual trials, 5 ms, *t* < 1. The three way interaction was due to this auditory global advantage being greater for within-modality, 93 ms, *t*(23) = 3.777, *p* = 0.001, Cohen’s *d* = 0.77, compared with across-modality transitions, 14 ms, *t*(23) = 2.133, *p* = 0.04, Cohen’s *d* = 0.44. In brief, an auditory global (over local) advantage was present overall, though most evident for within-modality transitions.

Lastly, a three-way interaction between level, modality priming and target priming emerged, *F*(1, 23) = 6.077, *p* = 0.022, η*_*p*_*^2^ = 0.21 (Figure 7). Across-modality, a global advantage was absent for repeated targets, 28 ms, *t*(23) = 1.604, *p* = 0.12, Cohen’s *d* = 0.33, but present for changed targets, 52 ms, *t*(23) = 2.669, *p* = 0.01, Cohen’s *d* = 0.55. The reverse was true for within-modality transitions: a global advantage was present for repeated targets, 44 ms, *t*(23) = 2.307, *p* = 0.03, Cohen’s *d* = 0.47, but absent (19 ms) for changed targets, *t* < 1, Cohen’s *d* = 0.18. No other higher order interactions reached significance.

## 4. Discussion

The primary result from this study is that no cross-modal attentional persistence for scope occurred—participants did not benefit from targets being presented locally (or globally) for subsequent local (or global) targets when switching from vision to audition, or vice versa (Figure 3). Surprisingly, and contrary to previous *unimodal* auditory and visual studies (e.g., Ward, 1982; Robertson, 1996; Filoteo et al., 2001; Justus and List, 2005; List and Justus, 2010; List et al., 2013), the multi-modal context also eliminated within-modality level-specific priming, as supported by the Bayes factor showing moderate evidence for the null hypothesis. Even when visual (or auditory) targets followed other visual (or auditory) targets, the typical benefit for repeating a target’s local or global level was absent. Thus, the multi-modal context interfered with unimodal attentional settings that typically drive persistence in processing local or global information. Paradoxically, the disruption of *unimodal* scope priming suggests that the multi-modal context has an impact on level-specific attentional persistence. These data are therefore inconsistent with fully independent visual and auditory attentional systems—were the systems entirely independent, cross-modal level-specific priming would not emerge, but within-modality level-specific priming should still manifest. The data are also inconsistent with the hypothesized level-specific cross-modal interactions, because none emerged. Instead, the data point to a goal-directed or strategic cross-modal interaction whereby maintaining attentional flexibility across modalities with distinct hierarchical levels has as its consequence the elimination of unimodal level-specific priming.

Critically, however, not all priming effects were eliminated. Identity priming (reflected as an advantage for repeated level vs. changed level in repeated target/response trials) was present in three cases, and the Bayes factor revealed very strong to extreme support for a difference between conditions. Namely, for visual trials, whether preceded by auditory or visual trials, participants showed a benefit for the target pattern to repeat at the same level rather than change levels. This was also true for within-modality auditory trials. These results are important because they establish the rigor of the method in detecting priming effects, whether for visual or auditory targets (whose RTs do differ considerably). These identity priming results suggest that the null level-priming effects are not simply due to, for example, poor execution or unmotivated participants—otherwise, neither would be present.

Because the absence of unimodal level-priming effects was unexpected, it is important to consider how the multi-modal context may have disrupted attentional persistence across modalities. One consideration is whether presenting stimuli in both visual and auditory modalities created an additional load on participants compared with prior unimodal studies. Indeed, participants were required to process more and different kinds of stimuli. However, a few points challenge a (simple) load argument. First, all participants were required to practice until meeting a uniform minimum level of accuracy within each modality and in a multi-modal context. Therefore, commensurate with previous unimodal auditory studies in which level-priming occurred (e.g., Justus and List, 2005; List and Justus, 2010), a baseline level of accuracy was achieved. Second, when comparing the accuracy rates and RTs from this study to other unimodal studies showing level-priming effects (e.g., Robertson, 1996; Kim et al., 1999; Justus and List, 2005; List and Justus, 2010; List et al., 2013), performance is well-matched for each modality. Third, the multi-modal context only affected level-priming, and not identity priming. Any argument that load-related difficulty eliminated priming effects would need to account for why identity priming would be spared, whereas level-priming effects would be *selectively* eliminated. Although the current study cannot rule out the possibility that other load manipulations might have similar selective consequences, it is at least established that when participants are required to allocate attention flexibly across visual and auditory scope, there is no evidence that they derive a benefit from repetition of attentional selection within or across modalities.

So why were cross-modal level-specific interactions not found? One possibility is that they will never occur. However, from previous studies (see, e.g., the debate between Spence and Driver, and Ward and his colleagues referenced in the Introduction), variations in stimulus and task parameters can substantively affect whether cross-modal effects are observed. It may yet be possible that level-specific attentional persistence across modalities might occur with variations in methodological approach. One candidate stimulus change is drawn from the work of Ivry and Robertson (1998) and Robertson and Ivry (2000). They surveyed a broad range of research on hierarchical processing, and proposed an information processing theory, the double filtering by frequency theory. The theory holds that an initial attentional selection of relevant frequency information occurs in both vision and audition, and that a subsequent second stage involves the attentional filtering of relatively higher and lower frequencies in left and right hemispheres, respectively. There is ample evidence that visual spatial frequency selection is what underlies, or at least depends on similar mechanisms as, attentional selection of local or global information (e.g., Shulman et al., 1986; Shulman and Wilson, 1987; Robertson, 1996; Flevaris et al., 2011). Furthermore, processing of auditory frequency information has been shown to reflect similar hemispheric asymmetries (Ivry and Lebby, 1993) to those engaged in processing visual spatial frequencies (e.g., Kitterle et al., 1990). Thus, to observe cross-modal level-specific priming, it may be important that the auditory and visual stimuli be better matched by both requiring frequency selection (e.g., using the stimuli from Justus and List, 2005, Experiment 1). In the current study, though local and global auditory selection *could* be based on frequency information, the patterns vary over time as well, and therefore participants could use both the frequency and temporal dimensions to make their decisions. Thus, in this study, it may be that this additional auditory temporal dimension interfered with cross-modal interactions that might otherwise occur when only frequency-based selection is possible. One compelling piece of evidence supports the importance of frequency selection in producing level-specific priming: Robertson (1996) manipulated the spatial frequency content in visual hierarchical stimuli, and showed that level-based priming effects were eliminated (whereas, importantly, they occurred under other stimulus manipulations).

Another possible avenue for future research into cross-modal hierarchical processing is to match auditory and visual stimuli on the basis of a spatial hierarchy. Some evidence points to the necessity that space be relevant in both modalities for cross-modal effects to emerge (e.g., Spence and McDonald, 2004). In the current study, space was only relevant in the visual modality, because by adopting Justus and List’s (2005, Experiment 2) stimulus set, this study inherently adopts their arguments that frequency and time are the relevant auditory dimensions for local and global selection (also relying indirectly on Kubovy and Van Valkenburg’s (2001) auditory object attributes). Although spatial locations may seem evidently analogous across vision and audition, in multi-modal research, a ubiquitous and persisting problem is understanding which dimensions might be analogous across modalities and how flexible these mappings are (e.g., Marks, 1974; Evans and Treisman, 2010). For instance, even though space is common to multiple modalities, visual space can map to multiple auditory dimensions (e.g., auditory space or frequency). Nevertheless, because auditory hierarchical stimuli varying in frequency and time did not interact with visual spatial hierarchical stimuli here, it would be worth further investigating cross-modal interactions with both auditory and visual *spatial* hierarchical stimuli—under those conditions, stimuli in both modalities would be able to be parsed spatially into local and global levels, potentially providing an even stronger opportunity for cross-modal interactions to arise.

## Data availability statement

The raw data supporting the conclusions of this article will be made available by the authors, without undue reservation.

## Ethics statement

The studies involving human participants were reviewed and approved by Hamilton College Institutional Review Board (SP14-112). The patients/participants provided their written informed consent to participate in this study.

## Author contributions

AL was responsible for the reported research and writing.
