# Supplementary material for: Global and local priming in a multi-modal context
Source: Front Hum Neurosci. 2023 Feb 28;16:1043475. doi: 10.3389/fnhum.2022.1043475 (PMC10011069; doi:10.3389/fnhum.2022.1043475)
Supplement: Supplementary file 1 [file Table_1.docx]

## Supplementary Table

| Variables | *F*(1, 23) | *p* | $\eta_{p}^{2}$ |
| --- | --- | --- | --- |
| **Modality (mod)** | **328.965** | **.000** | **.935** |
| *Level (level)* | *4.329* | *.049* | *.158* |
| **Modality Priming (modP)** | **39.709** | **.000** | **.633** |
| Target Priming (targP) | 2.408 | .134 | .095 |
| **Level Priming (levelP)** | **17.391** | **.000** | **.431** |
| **mod * level** | **17.249** | **.000** | **.429** |
| mod * modP | .001 | .976 | .000 |
| level * modP | .577 | .455 | .024 |
| *mod * level * modP* | *6.156* | *.021* | *.211* |
| mod * targP | .051 | .823 | .002 |
| level * targP | .005 | .946 | .000 |
| mod * level * targP | .075 | .787 | .003 |
| modP * targP | .582 | .453 | .025 |
| mod * modP * targP | 2.458 | .131 | .097 |
| *level * modP * targP* | *6.077* | *.022* | *.209* |
| mod * level * modP * targP | 2.922 | .101 | .113 |
| mod * levelP | .134 | .717 | .006 |
| level * levelP | 1.572 | .223 | .064 |
| mod * level * levelP | 1.717 | .203 | .069 |
| **modP * levelP** | **24.485** | **.000** | **.516** |
| *mod * modP * levelP* | *4.496* | *.045* | *.164* |
| level * modP * levelP | .545 | .468 | .023 |
| mod * level * modP * levelP | .295 | .592 | .013 |
| **targP * levelP** | **23.324** | **.000** | **.503** |
| mod * targP * levelP | 1.726 | .202 | .070 |
| level * targP * levelP | .287 | .598 | .012 |
| mod * level * targP * levelP | .803 | .380 | .034 |
| modP * targP * levelP | 2.006 | .170 | .080 |
| mod * modP * targP * levelP | .313 | .581 | .013 |
| level * modP * targP * levelP | .827 | .372 | .035 |
| mod * level * modP * targP * levelP | .679 | .418 | .029 |

*Note.* Omnibus 5-way ANOVA results. See text for description of reliable effects and interactions, as well as follow up comparisons for interactions. Emboldening indicates main effects or interactions which exceed Bonferroni or Holm-Bonferroni correction methods for multiple comparisons (which resulted in equivalent outcomes). Italicization indicates main effects or interactions which do not survive correction for multiple comparisons.
